# Mechanistic Scrutiny Identifies a Kinetic Role for Cytochrome b5 Regulation of Human Cytochrome P450c17 (CYP17A1, P450 17A1)

**DOI:** 10.1371/journal.pone.0141252

**Published:** 2015-11-20

**Authors:** Alexandr N. Simonov, Jessica K. Holien, Joyee Chun In Yeung, Ann D. Nguyen, C. Jo Corbin, Jie Zheng, Vladimir L. Kuznetsov, Richard J. Auchus, Alan J. Conley, Alan M. Bond, Michael W. Parker, Raymond J. Rodgers, Lisandra L. Martin

**Affiliations:** 1 School of Chemistry, Monash University, Clayton, Victoria, Australia; 2 ACRF Rational Drug Discovery Centre, St. Vincent's Institute of Medical Research, Fitzroy, Victoria, Australia; 3 School of Veterinary Medicine, University of California Davis, Davis, California, United States of America; 4 Department of Physiology and Membrane Biology, School of Medicine, University of California Davis, Davis, California, United States of America; 5 Boreskov Institute of Catalysis, Prospekt Lavrentieva 5, Novosibirsk, Russia; 6 Division of Metabolism, Endocrinology and Diabetes, Department of Internal Medicine, University of Michigan, Ann Arbor, Michigan, United States of America; 7 Department of Biochemistry and Molecular Biology, Bio21 Molecular Science and Biotechnology Institute, University of Melbourne, Parkville, Victoria, Australia; 8 Discipline of Obstetrics and Gynaecology, School of Paediatrics and Reproductive Health, Robinson Research Institute, University of Adelaide, Adelaide, South Australia, Australia; University of Colorado Boulder, UNITED STATES

## Abstract

Cytochrome P450c17 (P450 17A1, CYP17A1) is a critical enzyme in the synthesis of androgens and is now a target enzyme for the treatment of prostate cancer. Cytochrome P450c17 can exhibit either one or two physiological enzymatic activities differentially regulated by cytochrome b5. How this is achieved remains unknown. Here, comprehensive *in silico*, *in vivo* and *in vitro* analyses were undertaken. Fluorescence Resonance Energy Transfer analysis showed close interactions within living cells between cytochrome P450c17 and cytochrome b5. *In silico* modeling identified the sites of interaction and confirmed that E48 and E49 residues in cytochrome b5 are essential for activity. Quartz crystal microbalance studies identified specific protein-protein interactions in a lipid membrane. Voltammetric analysis revealed that the wild type cytochrome b5, but not a mutated, E48G/E49G cyt b5, altered the kinetics of electron transfer between the electrode and the P450c17. We conclude that cytochrome b5 can influence the electronic conductivity of cytochrome P450c17 via allosteric, protein-protein interactions.

## Introduction

Cytochrome P450 (P450) enzymes are ubiquitous throughout nature, utilizing a heme moiety at their active site with the electrons delivered *via* a protein redox pathway for hydroxylation of substrates [1]. Our current understanding of mammalian P450 biochemistry is derived mostly from studies on hepatic P450 enzymes. These differ from steroidogenic P450s in two fundamental ways. The hepatic P450s typically catalyze mono-hydroxylations needed to solubilize xenobiotics, required to assist with their excretion from the body. They have large, flexible active site cavities that accommodate numerous substrates. In comparison, the steroidogenic P450s catalyze multiple hydroxylations and carbon-carbon bond cleavage. Also, the active site cavities are typically much smaller and accommodate only a narrow range of substrates. In addition, the activities of both groups of P450s require different ratios of the redox partner protein, NADPH-cytochrome P450 oxido-reductase (CPR). The ratio of P450 to CPR in hepatic tissues is large whereas in steroidogenic tissues it is believed to be much lower [2–4].

The steroidogenic enzyme cytochrome P450 17α-hydroxylase (P450c17; encoded by *CYP17A1*) is an enzyme needed to synthesize both androgens (male hormones) and glucocorticoids (*e*.*g*. cortisol, essential for metabolism) from pregnenolone [5, 6]. P450c17 has a single enzymatic activity, 17α-hydroxylase, in the zona fasciculata of the adrenal gland, which produces cortisol. However, it has two activities, 17α-hydroxylase and 17,20-lyase, in the gonads and in the adrenal zona reticularis. These two activities are essential for the conversion of pregnenolone to dehydroepiandrosterone (DHEA), required for androgen synthesis. The first enzymatic activity, 17α-hydroxylase, is a single hydroxylation and the second requires an additional hydroxylation leading to the breaking of the bond between carbons 17 and 20 [7]. Therefore, both activities of P450c17 require electrons from NADPH delivered *vi*a CPR.

Close examination of the cellular locations of P450c17 revealed that the gonads and the adrenal zona reticularis are also rich in cytochrome b5 (cyt b5) [8–10], a small redox protein which is co-located with P450c17 [9]. Cyt b5 has been implicated in promoting the synthesis of androgens at the expense of cortisol production [11]. Cyt b5 is a common electron transfer (redox) protein; however, apo-cyt b5 with no redox activity also enhances androgen synthesis, suggesting that cyt b5 has an allosteric interaction with P450c17, not a redox interaction [12]. We have shown previously that a double mutant E48G/E49G cyt b5 was unable to stimulate the 17,20-lyase activity of P450c17 [12]. It has also been shown that cyt b5 interacts directly with P450c17 [4, 13, 14] and recently, using the soluble domain of cyt b5, that the interaction site on P450c17 is shared with CPR [4, 15]. Previous research in this area has indicated that cyt b5 does not alter the active site of P450c17 as the Michaelis constant and substrate binding affinity were unchanged [16]. Nonetheless, it remains unknown just how cyt b5 regulates the enzymatic activity of P450c17. Recent work has benefited considerably from the crystal structure of P450c17 [17], however, despite this structural information questions remain unanswered as to the specific protein-protein interactions and regulation of androgen synthesis by P450c17. Here we investigate the protein-protein interactions to identify how cyt b5 regulates the 17,20-lyase activity of P450c17; we have conducted studies using *in silico* docking analysis, fluorescence resonance energy transfer (FRET) studies, quartz crystal microbalance (QCM) analyses and electrochemical studies. Together these data show that the cyt b5 interacts with P450c17 via a well-defined allosteric binding site and dynamically regulates the electrical conductivity of P450c17.

## Materials and Methods

### Materials

High-purity water (18 MΩ∙cm; Sartorius Arium 611) was used for all procedures. KH_2_PO_4_ (SigmaAldrich; ≥ 99 wt.%), K_2_HPO_4_ (SigmaAldrich; ≥ 99.9 wt.%), NaCl (Fluka, Ultra, > 99.5 wt.%), H_3_BO_3_ (Ajax Finechem), H_2_SO_4_ (Univar; 98 wt.%), H_2_O_2_ (Merck, Emsure; 30 wt.% aqueous solution), NH_4_OH (Ajax Finechem, 28 wt.% aqueous solution), NaOH (SigmaAldrich; > 97 wt.%), isopropanol (Merck, Epmarta; ≥ 99.5%), chloroform (SigmaAldrich; ≥ 99%), ethanol (Merck, Emsure; absolute), acetone (Merck, Emplura; > 99%), acetonitrile (≥ 99.9%, Merck LiChrosolv), methyl *tert*-butyl ether (Sigma 99.8%), glycerol (Sigma), mercaptopropionic acid (Fluka, BioChimica; ≥ 99.0%), hexanethiol (Fluka; ≥ 95%), 1,2-dimyristoyl-*sn*-glycero-3-phosphocholine (DMPC) (Avanti polar lipids), cholesterol (SigmaAldrich, ≥ 99.0%), polymyxin B sulphate salt (Sigma; 7870 units g^-1^), hemin (Sigma; porcine, ≥ 98%,), pregnenolone (Steraloids, USA), 17α-hydroxy-pregnenolone (Steraloids, USA), dehydroepiandrosterone (Steraloids, USA), N,O-bis(trimethylsilyl) trifluoroacetamide (SigmaAldrich; ≥ 99.0%), trimethylchlorosilane (SigmaAldrich; ≥ 99.0%), carbon paper (AvCarb P50) were used as received from the manufacturers. Dichloromethane (MERCK Suprasolv; > 99.9%) was distilled over CaH_2_ under a N_2_ atmosphere prior to use. High-purity nitrogen (99.999%, O_2_ < 2 ppm) was used to remove oxygen from the working electrolyte solutions in voltammetric studies when required.

Tissue culture materials were purchased from Invitrogen, Fisher, and Sigma. CFP (eCFP-N1) and YFP (eYFP-N1 and eYFP-C1) vectors were purchased from Clontech (USA). Solutions of the wild type (wt) and mutant (E42G, E74G, R52G, E48/49G) human cyt b5 were purified as reported elsewhere [12]. Solutions of recombinant cytochrome P450c17 and CPR were purified as reported elsewhere [2, 18]. All protein solutions were stored at -80°C in small aliquots (50–200 μL) and thawed on ice prior to use.

Multiwall carbon nanotubes (CNT; 22 nm average diameter) with impurities less than 1 ppm, were synthesized using a Fe-Co catalyst, boiled in HCl, annealed at 2800°C in a high-purity Ar atmosphere and characterized following established procedures [19, 20]. The CNT samples were not ball milled so as to exclude disruption of the regularity or formation of additional defects on the carbon ‘walls’. Transmission electron microscopic images in Figure A in S1 File show the fine structure of the heat-treated CNTs.

### FRET fusion constructs

The cDNAs encoding human P450c17 and cyt b5 were engineered so as to generate proteins fused with eYFP or eCFP at the C-terminus, or for cyt b5 at the N-terminus. P450c17 construct was made using the eCFP-N1 vector to make the fluorescent protein as a C-terminal fusion because the N-terminus of P450c17 encodes the trans-membrane domain. The cyt b5 construct was made using eYFP-C1 vector to make a N-terminal fusion because cyt b5 is membrane anchored at its carboxy-terminus. The CPR-eYFP C-terminal fusion construct was donated generously by Dr. Byron Kemper (University of Illinois), and its successful use in FRET experiments has been described previously [21]. The successful expression of the fusion constructs was confirmed by western immunoblotting, which demonstrated the expected increase in molecular size for P450c17 as previously reported [22] and for cyt b5 (Figure B in S1 File). Furthermore these fusion proteins were catalytically active as illustrated in Figure C in S1 File.

### Transfection into cell lines

Transfection of HEK293 was conducted in HyClone (Logan, UT) media with fetal bovine serum. HEK293 cells (American Type Culture Collection, Manassas, VA) were cultured on Primaria cultureware (BD Biosciences, San Jose, CA) in Dulbecco's Modified Eagle’s Medium with 5% fetal bovine serum, 10 mM HEPES (pH = 7.4), 100 U ml^-1^ penicillin, and 100 μg ml^-1^ streptomycin. After 24 h, cells were transfected (4 μg / 35 mm well) with plasmid fusion constructs using Lipofectamine 2000 (Invitrogen, Carlsbad, CA) as suggested by the manufacturer. For FRET experiments, transfected cells were plated after 18 h into Lab-Tek Chambered Coverglass (1.0 borosilicate coverglass; Nunc, Rochester, NY) at 40% confluence and cultured for 24 h at 37°C.

### Analysis of FRET

FRET was evaluated as previously described from the enhanced acceptor emission [22, 23]. HEK293 cells were transfected with P450c17-eCFP and b5-eYFP plasmids and replated into Lab-Tek Chambered Coverglass 24 h later. Fluorescence and spectral images (from multiple cells and multiple experiments) were captured on a computer-controlled, inverted Olympus IX-81 epifluorescence microscope (Olympus USA, Center Valley, PA) fitted with an Acton SpectraPro215 spectrograph (Roper Scientific, Tucson, AZ). A mercury light source was used for excitation in conjunction with filter cubes, containing an excitation filter and dichroic mirror (CFP: excitation filter-D436/20, dichroic-455DCLP; YFP: excitation filter-HQ500/20, dichroic-Q515LP). Two spectral images of each individual cell, expressing either the CFP construct, the YFP construct, or co-expressing both constructs, were recorded using CFP and YFP excitation cubes, respectively. FRET data included measurements from 15 different cells taken from experiments run on 7 different days, each including 1–4 cell recordings. Fluorescence emission spectra were constructed from spectral images after background subtraction and analyzed using MetaMorph (Molecular Devices Corp., Sunnyvale, CA) in conjunction with Microsoft Excel. FRET ratios were calculated from these spectra.

### Structural modeling

Chain A from the crystal structure of Human Cyt P450 17A (PDB code:3swz) [17] was initially docked onto model 1 of the solution structure of human cyt b5 (PDB code:2i96) [24] using ZDOCK 3.0.2 [25]. All parameters were kept at default. The 20 lowest energy solutions were then obtained and visually analyzed. In this analysis, 60% of the solutions showed a similar binding mode, thus the lowest energy complex from this cluster was chosen for further studies. Heme was removed from this complex and then it was submitted to RosettaDock [26] under default docking parameters. The output energies are listed in Table A in S1 File. The top 10 scoring solutions were obtained and visually analyzed. There was one major cluster (70%) of solutions, with the highest scoring of these the second lowest energy solution. This complex was kept for further analysis. Visual analysis was undertaken and figures were constructed using the PyMOL Molecular Graphics System, Version 1.6.0.0 Schrödinger, LLC.

### Molecular dynamics simulations

The molecular dynamics program NAMD [27] was used to minimize the P450c17:cyt b5 complex. This complex was initially solvated with TIP3P water using the Solvate plugin within VMD version 1.9. The rotate to minimize volume was selected, the boundary reduced to 1.8 and the box padding increased to 20 Å in all directions; all other parameters were kept at default. Charges were then neutralized with NaCl using the autoionize plugin within VMD version 1.9 using default settings. Each structure was minimized and equalized for 1 nanosecond (2 femtosecond time step) under the CHARMM27 all-atom force field at 298 K. Langevin dynamics were used with group pressure and Langevin piston were turned on. Trajectory snapshots were collected every picosecond. After approximately 200 picosconds, the average RMSD had leveled to approximately 2 Å and analysis was conducted after this time point. Hydrogen bond occupancy was calculated using the Hydrogen Bond plugin in VMD version 1.9 with a donor-acceptor distance at 3.0 Å and an angle cutoff of 20°. Detailed information was then selected to be included in the calculation for ‘All H-bonds’.

### Quartz crystal microbalance procedures

QCM measurements were undertaken using a Q-sense E4 instrument (Sweden) and quartz crystal disks coated with a thin Au layer (Q-Sense; diameter *ca* 1 cm; fundamental resonant frequency 5 MHz) mounted into a cell thermostated at 295 K. This temperature was selected based on the transition temperature of the lipids used to create a membrane layer, as well as the lowest temperature to avoid nano-bubbles that could cause difficulties in the QCM apparatus. Prior to use, gold-coated quartz crystals were cleaned in a NH_4_OH (28 wt.% aqueous solution):H_2_O_2_ (30 vol.% aqueous solution):H_2_O (1:1:3 vol.) mixture at 70°C for 15 min, rinsed plentifully with water and isopropanol, and immersed in an isopropanol solution of 1.0 mM mercaptopropionic acid (mpa) for at least 30 min for formation of a firmly bound mpa layer. Further, crystals were carefully washed with isopropanol to remove unbound mpa, dried under a gentle stream of nitrogen and installed in a QCM cell. An Ismatec peristaltic pump (ISM935; Switzerland) was used to introduce solutions into the cell. The changes in frequency (Δ*f*_QCM_) and dissipation (Δ*D*) associated with the third, fifth, seventh, and ninth harmonics were recorded during the experiment, but changes in 7^th^ overtone only are presented and discussed below (frequency data are reported as normalized Δ*f*_QCM_/7 values). After attaining a stable QCM response in water, 0.1 M NaCl + 0.02 M (K_2_HPO_4_+KH_2_PO_4_) (pH = 6.9) solution was pumped through the cell at the flow rate of 0.30 ml min^-1^ until stable Δ*f*_QCM_ and Δ*D* values were attained. Further, lipid membranes were created on top of the mpa monolayer attached to the Au-coated quartz crystals by depositing liposomes of 1,2-dimyristoyl-*sn*-glycero-3-phosphocholine (DMPC) containing 20 mol.% cholesterol from the 0.1 mM liposome solution in 0.10 M NaCl + 0.02 M (K_2_HPO_4_+KH_2_PO_4_) (pH = 6.9) pumped through the cell at 0.10 ml min^-1^ for 15–25 min following the previously documented protocol [28]. Lipid deposition was followed by rinsing the cell with 0.04 M NaCl + 0.008 M (K_2_HPO_4_+KH_2_PO_4_) (pH = 6.9) solution at 0.30 ml min^-1^ for 10–15 min to promote removal of unopened liposomes [29] and then re-introduction of the 0.10 M NaCl + 0.02 M (K_2_HPO_4_+KH_2_PO_4_) (pH = 6.9) environment at 0.30 ml min^-1^ until a stable QCM response was obtained. Finally, *ca* 1 ml of the protein(s) solution in 0.10 M NaCl + 0.02 M (K_2_HPO_4_+KH_2_PO_4_) (pH = 6.9) was pumped through the cell at 0.05 ml min^-1^. To allow QCM analysis of the structural changes occurring in the lipid layer upon deposition of protein mixtures over a longer period of time, the outlet of the QCM cell was connected to the inlet after the whole volume of the protein sample was pumped into the apparatus, so that the protein-containing solution was continuously circulated through the cell at 0.05 ml min^-1^ for as long as required.

### Electrochemical instrumentation and procedures

Electrochemical measurements were undertaken using either a BAS Epsilon electrochemical workstation (USA) or a custom-made Fourier transform a.c. voltammetric instrument (Monash University and LaTrobe University, Australia) [30] in three-electrode cells at ambient temperature (23 ± 1°C). Prior to use, all glassware was filled with the H_2_SO_4_ (98 wt.%):H_2_O_2_ (30 vol.%) (1:1 vol.) mixture for at least 12 hours, repeatedly washed with ultrapure water and dried in an oven at 110–120°C. To reduce uncompensated resistance (*R*_u_), aqueous 0.20 M NaCl + 0.02 M (K_2_HPO_4_+KH_2_PO_4_) (pH = 7.0) was used as the working electrolyte solution in all electrochemical experiments.

The high surface area Pt wire (voltammetry) or Pt mesh (controlled potential electrolysis) auxiliary electrode was immersed in the solution containing electrolyte and separated from the working electrode compartment by a glass frit. The reference electrode was a AgCl-coated Ag wire placed in a Luggin capillary also in a solution with electrolyte. This low-impedance reference electrode is advantageous for a.c. voltammetric measurements. The potential of this reference electrode (Ag|AgCl|0.20 NaCl + 0.02 M (K_2_HPO_4_+KH_2_PO_4_), pH = 7.0) did not vary between experiments and was 0.078 ± 0.001 V *vs*. Ag|AgCl|KCl(sat.) (*cf*. the theoretical value of 0.080 V) [31]. All potentials are reported versus Ag|AgCl|KCl(sat.) (0.197 V *vs*. normal hydrogen electrode) unless otherwise stated.

In the voltammetric studies, gold (nominal diameter 0.2 cm), or glassy carbon (GC; nominal diameter 0.3 cm) macrodisk electrodes (supplied by BAS (USA)), or a home-made pyrolytic graphite edge (PGE) or basal plane (PGB) square electrodes (*ca* 0.2 × 0.2 cm) embedded in an isolating inert sheath were used as the working electrodes. Prior to use, the surface of the working electrode was polished with Al_2_O_3_ powder (Buehler; 1 μm for PGE and PGB electrodes, 0.3 μm for Au and GC electrodes) using a wet polishing cloth (BAS), then washed, several times, with water, sonicated in water for *ca* 20 s (FXP 10M, U-LAB Instruments, Australia), wiped at least 50 times with a clean wet polishing cloth free of Al_2_O_3_, sonicated for another *ca* 20 s in a fresh portion of H_2_O, then finally washed again with water. In bulk electrolysis experiments, AvCarb P50 carbon paper (square, *ca* 0.5 × 0.5 cm) modified with CNTs was used as a working electrode.

### Fabrication of CNT-based electrodes

Deposition of a thin CNT layer on a clean and dry GC electrode surface (*ca* 30–45 μg_CNT_ cm^-2^) was performed by pipetting small aliquots (*ca* 0.3 μl) of a suspension of CNTs in isopropanol (0.10–0.20 mg ml^-1^) while under a gentle N_2_ flow to effect rapid drying. Preparation of the CNT-modified carbon paper electrode (350–400 μg_CNT_ cm_geom._^-2^) was undertaken similarly using larger aliquots (10–20 μl) of the CNT suspension on both sides of the electrode. Prior to their use, 0.1–0.2 mg ml^-1^ suspensions of the employed carbon nanotubes in isopropanol were sonicated for at least 45 min to attain good homogeneity, as exemplified in the photograph in Figure D in S1 File. However, rapid aggregation of the CNTs occurred after the sonication ceased. The suspensions were therefore continuously sonication during deposition.

Use of isopropanol did not allow for the selective deposition of the CNT suspension only on the GC surface, but extended over both conducting carbon surface and the isolating sheath, even if the smallest aliquots (< 1 μl) were applied to the electrode. Therefore, a CNT layer was deposited over the whole top surface of an electrode, and the CNTs that deposited on the isolating sheath were carefully removed using filter paper until only CNTs on a GC surface remained (Figure D in S1 File).

The high degree of hydrophobicity of the CNTs surface, due to the near perfect structure, was assessed by the poor wettability of the electrode surface in aqueous solutions. The CNT surface was made more hydrophilic by dipping the fabricated CNT/GC electrodes into aqueous glycerol solution (10 vol. %) for 10 min, then washed with water and stored in pure H_2_O for not more than 12 h until the experiment.

### Fabrication of Au electrodes modified by hexanethiol self-assembled monolayer

Prior to the deposition of self-assembled monolayers on a Au electrode surface, the electrodes were cleaned both ‘chemically’ and ‘electrochemically’. First, the electrode was kept in a H_2_SO_4_ (98 wt.%): H_2_O_2_ (30 vol.%) (1:1 vol.) mixture for at least 12 h; then the Au electrode was used as a working electrode for multiple cyclic voltammograms (0.020 V s^-1^) in deaerated aqueous 0.50 M H_2_SO_4_ solution over a potential range, 0.05 to 1.6 V *vs*. reversible hydrogen electrode (platinized platinum wire in contact with H_2_-saturated 0.50 M H_2_SO_4_) until a stable voltammetric response typical of pure polycrystalline gold was obtained.

Modification of the Au electrodes with a self-assembled monolayer of hexanethiol was undertaken by dipping a cleaned electrode in the 1–10 mM hexanethiol in isopropanol solution for 30 min and subsequently washing with copious amounts of pure isopropanol and then water.

### Protein adsorption and characterization of the electrodes

Protein-free electrodes were characterized electrochemically using the deoxygenated or air-saturated electrolyte solutions as appropriate. Protein samples were adsorbed on the electrode surface, over 15–60 min, under ambient conditions from a small aliquot (4–10 μl) of the stock protein solution. For polymyxin B-assisted adsorption of cyt b5 on PGE electrodes, protein solutions containing *ca* 0.13 mM polymyxin B were used and polymixin B was also present in the electrolyte solution. The protein-modified electrodes were placed in an electrochemical cell, and the electrolyte solution was deoxygenated by purging with nitrogen for 20 min. In all measurements, a nitrogen atmosphere was maintained in the cell by slowly purging the gas above the solution. In some experiments, the voltammetrically characterized protein-modified electrode was used for further experiments; either for adsorption of another protein and subsequent electrochemical characterization, or to test for the catalytic reduction of oxygen in an air-saturated electrolyte.

### Voltammetric simulations

Simulations of voltammograms were performed using the Monash Electrochemistry Simulator (MECSim) software [32].

### Gas chromatographic analysis

Preparation of the samples for the semiquantitative analysis of pregnenolone (preg) and 17α-hydroxypregnenolone (17-OH-preg) in aqueous solutions included the following procedures: (i) extraction of the analysed compounds from an aqueous phase to dichloromethane or methyl *tert*-butyl ether; (ii) evaporation of the organic solvent under gentle N_2_ flow and dissolution of the solids in CH_3_CN; (iii) trimethylsilylation of the target compounds with N,O-bis(trimethylsilyl)trifluoroacetamide (BSTFA) in the presence of 1 vol.% (with respect to BSTFA) trimethylchlorosilane (TMCS) at 65°C during 30 min (volume proportions were as follows: 0.05 ml of the CH_3_CN solution + 0.05 ml BSTFA with TMCS). Subsequently, gas chromatographic analysis with flame-ionization detector was undertaken under the conditions reported by Bowden *et al*. [33]. The derivatization conditions employed were based on results from Bowden *et al*. [33].

## Results

### *In vivo* FRET

**Fig 1:**
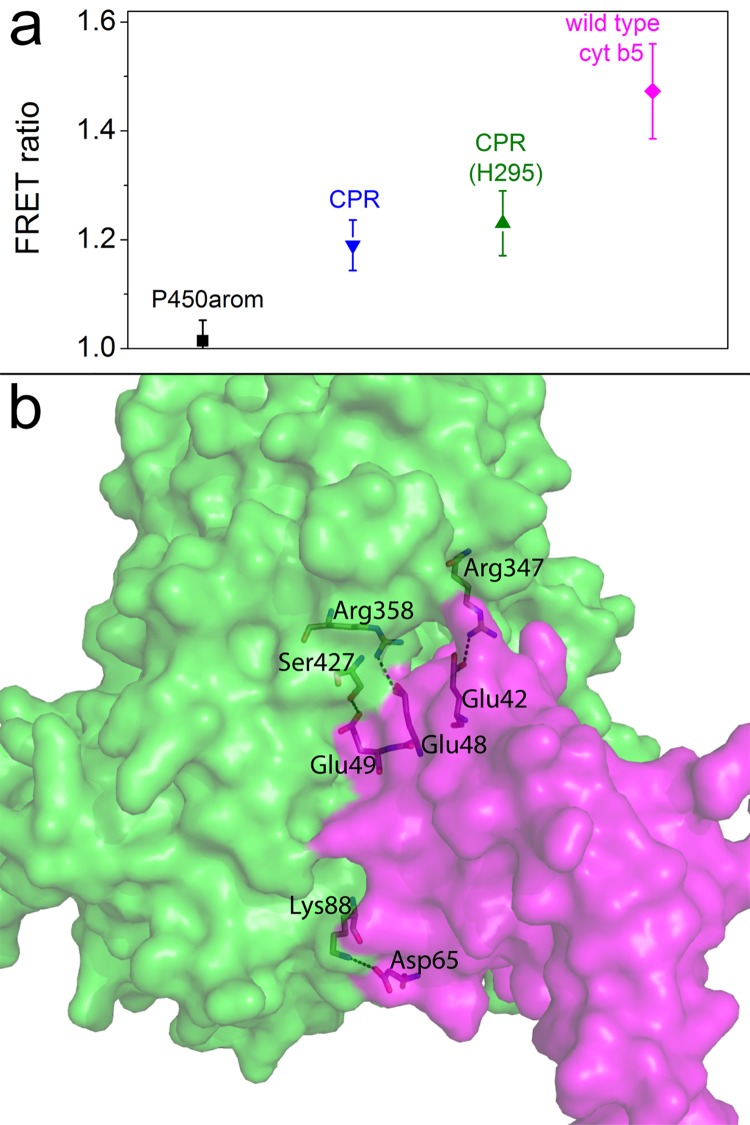
FRET ratio data for P450c17 and cyt b5 or CPR. **a,** The results of FRET pairs expressed in HEK293 or H295 (CPR, H295) cells. Fusion constructs P450c17-eYFP+CPR-eCFP (▲ and **▼**; positive controls) and P450c17-eYFP+P450arom-eCFP (■), where P450arom is a negative control.^17^ Importantly, the P450c17-eYFP+cytb5-eCFP (♦) have the highest FRET ratio. **b,** Model of the complex between P450c17 (green) and cyt b5 (magenta) shown in rendered style. Polar interactions are shown as black dashed lines. Those residues involved in polar interactions are highlighted and labeled individually.

Analysis by FRET of the *in vivo* protein-protein interactions between P450c17 with CPR using recombinant constructs to express these proteins in mammalian cells has been previously reported by some of us [22]. Here we examined the association between P450c17 and wild type (wt) cyt b5, comparing it with the association of P450c17 and CPR. Non-association of P450c17 with another steroidogenic cytochrome P450 aromatase (P450arom) was used as a negative control (Fig 1A). These protein-protein interactions were tested in steroidogenic (H295) and non-steroidogenic (HEK) cell lines with a similar response. A FRET ratio of >1.0 indicates close association and the apparent FRET efficiency for interaction of P450c17 with wt cyt b5 was significantly greater than with CPR, a clear demonstration of the close association (heterodimerization) between these two proteins *in vivo* (Fig 1A).

### Structural analysis of the protein-protein interaction sites

Structural analysis of the most likely binding interface between P450c17 and cyt b5 was examined via computational protein-protein docking. Fig 1B and Figure E in S1 File show that the P450c17—cyt b5 binding interface is composed of four putative polar interactions between cyt b5 residues Glu48, Glu49, Glu42 and Asp65 to P450c17 residues Ser427, Arg358, Arg347 and Lys88, respectively. This agrees well with a recent model by Peng *et al*. [4], with one additional interaction between Ser427 and Glu49 postulated in our model. They also reported cross-linking data supporting the close electrostatic interface shared by these two proteins. The specific cross-linked sites were Lys88-Glu61 and Lys347-Glu42 residues, connecting P450c17 and cyt b5, respectively [4]. The importance of Glu48 and Glu49 was revealed by mutagenesis with the double mutant E48G/E49G cyt b5 unable to stimulate the 17,20-lyase activity of P450c17 [12]. This study also suggested that mutation of Glu42 and Asp65 to glycine had an effect on the stimulation of 17,20-lyase activity, proposed to be due to a reduction in affinity between P450c17 and cyt b5. Therefore our docking solution agrees well with the previously reported 17,20-lyase activity data. Interestingly, 48 and 49 glutamate residues of cyt b5 were not found to be cross-linked [4] although clearly they are critical for the androgen synthesis pathway of P450c17. A further six cyt b5 amino acid mutations that did not influence 17,20-lyase activity were all remote from this predicted binding interface (Figure F in S1 File) [12].

Notably, mutagenesis of Arg358 and Arg347 amino acids on the P450c17 have been found to be clinically relevant, and subsequent studies have confirmed that these mutants selectively diminish 17,20-lyase activity whilst maintaining most of the 17α-hydroxylase activity [34]. Thus, these P450c17 residues are clearly important for interaction with cyt b5. Recent studies using solution NMR suggested that three arginine residues of P450c17 are important for binding to cyt b5 [15, 35]. The first two are Arg347 and Arg358, as described above, and the third residue was Arg449. Although this residue was not directly involved in polar interactions for the static protein-protein dock conducted in our experiments, the side chain of the Arg449 residue lies in between Arg 358 and Arg347 (Figure G in S1 File). Thus, we conducted a molecular dynamics simulation to explore this further. Notably, during the 100 ns simulation, the side chain of Arg449 was involved in a polar interaction with Glu48 approximately 53% of the time (Table B in S1 File). Thus it is conceivable that in a fluid protein environment, this amino acid would also play a role in stabilizing the protein-protein interface [15, 35].

### Quartz crystal microbalance studies

Specific protein-protein interactions in a physiologically relevant membrane are difficult to achieve. We have previously used a biomimetic 1,2-dimyristoyl-*sn*-glycero-3-phosphocholine (DMPC)-cholesterol composite lipid layer to measure specific protein-lipid and protein-protein interactions for P450c17 and CPR proteins using a QCM [22]. In these studies, solutions of each purified protein were found to bind tightly to the membrane resulting in a decrease in the normalized QCM frequency change (Δ*f*_QCM_ was negative on binding) versus time. The reproducibility for each individual protein was very good with the introduction of a P450c17 solution showing a Δ*f*_QCM_ about three times greater than with CPR [22] (data not shown), despite the distinctly different molecular weights, ~55 and 75 kDa, respectively. Significantly, addition of CPR to a membrane layer containing P450c17 showed strong binding, indicative of a specific interaction or heterodimerization between these two proteins [22]. For more details on the protein-lipid interactions see references [22, 28].

**Fig 2:**
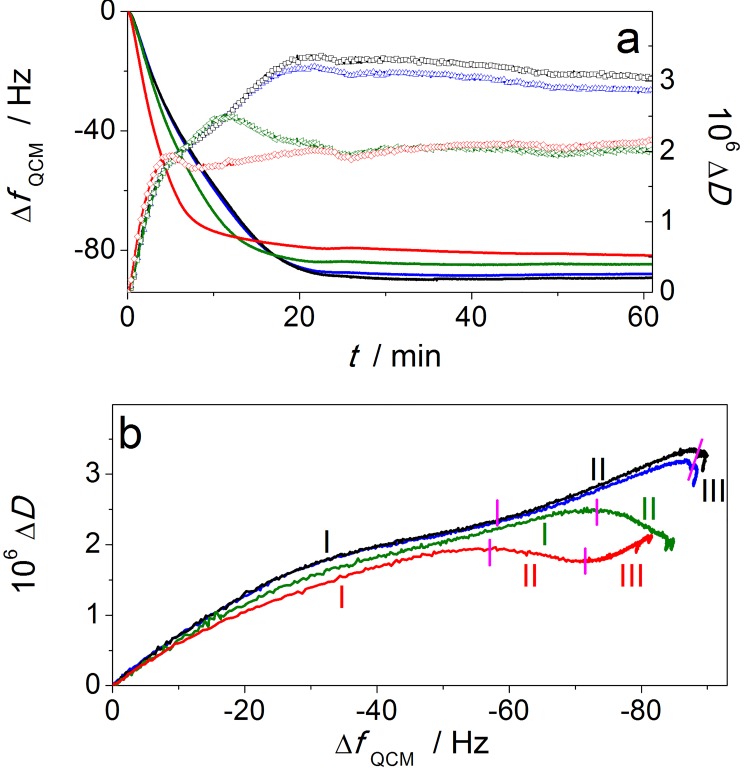
Quartz Crystal Microbalance data for proteins binding to a membrane layer. **a,** QCM data showing changes in frequency (lines, left axes) and dissipation (symbols, right axes) (7^th^ harmonic; Δ*f*_QCM_ data are normalized to the overtone number) derived from deposition of proteins onto a DMPC-cholesterol membrane pre-deposited on a mpa-layer adhered to a Au-coated quartz crystal. QCM profiles obtained with protein mixtures 20 nM P450c17+20 nM CPR+20 nM wt cyt b5 (blue) and 20 nM P450c17 + 20 nM CPR + 100 nM wt cyt b5 (black) were similar to each other and also to that for 20 nM P450c17+20 nM CPR (Figure H in S1 File). In contrast, the E48G/E49G cyt b5 was deposited non-specifically and competitively with P450c17+CPR, as determined with 20 nM P450c17+20 nM CPR+20 nM E48G/E49G cyt b5 (green) and especially 20 nM P450c17+20 nM CPR+100 nM E48G/E49G cyt b5 (red) mixtures. **b,** Analysis of the QCM data using Δ*f*_QCM_ *vs*. Δ*D* ‘fingerprint’ plots of the temporally resolved data from panel (a). The traces were divided into characteristic deposition stages labeled with roman numerals (I–III). DMPC = 1,2-dimyristoyl-*sn*-glycero-3-phosphocholine; mpa = mercaptopropionic acid.

In the current study we introduced equimolar mixtures of P450c17 and CPR in the presence or absence of the wt cyt b5 to the DMPC-cholesterol membrane layer and monitored temporal Δ*f*_QCM_ and dissipation change (Δ*D*) profiles as shown in Fig 2A and Figure C, panels a-c in S1 File. The changes in the QCM frequency and dissipation were essentially identical during all deposition stages (Fig 2A), irrespective of an equimolar or five-fold excess of wt cyt b5. Qualitatively these data also resembled profiles for P450c17 solutions binding at a DMPC-cholesterol membrane [22]. It is noteworthy that a solution containing only the wt cyt b5 also bound tightly on a DMPC-cholesterol layer, where monotonic changes in Δ*f*_QCM_ and Δ*D* characterized the protein binding to the membrane (Figure H, panels d and e in S1 File). The QCM data for P450c17 with CPR mixtures containing different amounts of wt cyt b5 (1:1:1 or 1:1:5 ratio) showed similar Δ*f*_*QCM*_–Δ*D*-*t* profiles and were primarily defined by the interaction of P450c17 (or a P450c17-CPR complex) with the lipid membrane (Fig 2A). Thus the deposition of wt cyt b5 protein contributed negligibly to the overall QCM profiles in terms of both total amount of protein deposited (reflected in the Δ*f*_QCM_-*t* data) and the properties of the ensuing protein-membrane layer (reflected in the Δ*D*-*t* data).

A fundamentally different situation was found for mixtures of P450c17 and CPR containing different concentrations of the E48G/E49G cyt b5 double mutant. Under these conditions, the rate of Δ*f*_QCM_ decrease during the first *ca* 5–10 min of interaction was significantly enhanced, although the total mass of proteins deposited decreased slightly with an increase in the E48G/E49G cyt b5 concentration (see Fig 2B and Figure H, panels b and c in S1 File). Changes in the viscoelastic properties of the protein-lipid layer induced by the presence of E48G/E49G cyt b5 were manifested by differences in the Δ*D*(*t*) profiles and final dissipation values were lower for mixtures of P450c17 + CPR containing the E48G/E49G cyt b5 than solution containing the wt cyt b5 (Fig 2A and Figure H, panel a in S1 File). The most probable explanation is that the QCM response arose from the competing deposition of uncoupled (individual) E48G/E49G cyt b5 and P450c17-based structures or complexes onto the lipid membrane. Thus, the double mutant cyt b5 bound the membrane more rapidly in sharp contrast to the wt cyt b5 with P450c17 + CPR solutions. The differences between P450c17 + CPR + wt cyt b5 and P450c17 + CPR + E48G/E49G cyt b5 mixtures revealed by QCM cannot be readily explained by different modes of interaction of individual solutions of wt cyt b5 and E48G/E49G cyt b5 with DMPC-cholesterol composite layer, as those were found to be essentially similar (Figure H, panels d-f in S1 File).

The QCM data for the mixtures of P450c17 + CPR containing either wt cyt b5 or E48G/E49G cyt b5 can be rationalized if there was specific coupling between wt cyt b5 and P450c17 (or P450c17-CPR complex). Indeed, based on the QCM data (Fig 2 and Figure C in S1 File), wt cyt b5 appeared to be incorporated in a ‘protein complex’ structure, in which the interaction with the lipid membrane was determined by the comparatively large P450c17 enzyme (or even larger P450c17-CPR complex) and was not influenced by the small cyt b5 protein. This rationalization also explains the QCM behavior for the E48G/E49G cyt b5 with P450c17 (or P450c17-CPR complex) with the membrane due to the competition of unbound E48G/E49G cyt b5. Furthermore, the effect was more marked with an increase in the E48G/E49G cyt b5 concentration.

Supporting the pronounced kinetic differences for protein(s) deposition discussed above, analysis of these data using Δ*f*_QCM_ *-*Δ*D* profiles compared the structural changes during protein deposition in terms of stages (see Fig 2B). Importantly, these stages arose during the protein(s) solution cycling through the QCM chamber and thus reflect the conformational evolution of the protein-lipid configurations (see Figure H in S1 File). In particular, there were several clear distinctions in the Δ*f*_QCM_ *-*Δ*D* profiles between solutions containing wt cyt b5 versus E48G/E49G cyt b5, in stages II–III (Fig 2B). The clearest effect was observed during stage III for a higher concentration of E48G/E49G cyt b5 in a mixture with P450c17 and CPR. The steady increase in mass (decrease in Δ*f*_QCM_), together with a sustained increase in Δ*D* was apparent and consistent with slow non-specific deposition of E48G/E49G cyt b5 on top of the protein-lipid layer (Fig 2B red trace). Importantly, this contrasted with the P450c17 + CPR + wt cyt b5 mixtures in which no deposition occurred as the mixtures of wt cyt b5 specifically bound to P450c17 + CPR from the initial solution (Fig 2B black trace). In fact, the negligible changes in Δ*f*_QCM_ and the small decrease in Δ*D* for the wt cyt b5 mixtures reflect the improved protein organization of the membrane layer gleaned by an overall increased rigidity of the surface.

In summary, QCM studies on the protein-lipid interactions with the P450c17 + CPR mixtures containing different ratios of wt cyt b5 or E48G/E49G cyt b5 confirmed the crucial importance of the glutamic acid residues 48 and 49 for a structural integration of the cyt b5 interaction with P450c17 (or P450c17-CPR complex).

### Electrochemical studies

**Fig 3:**
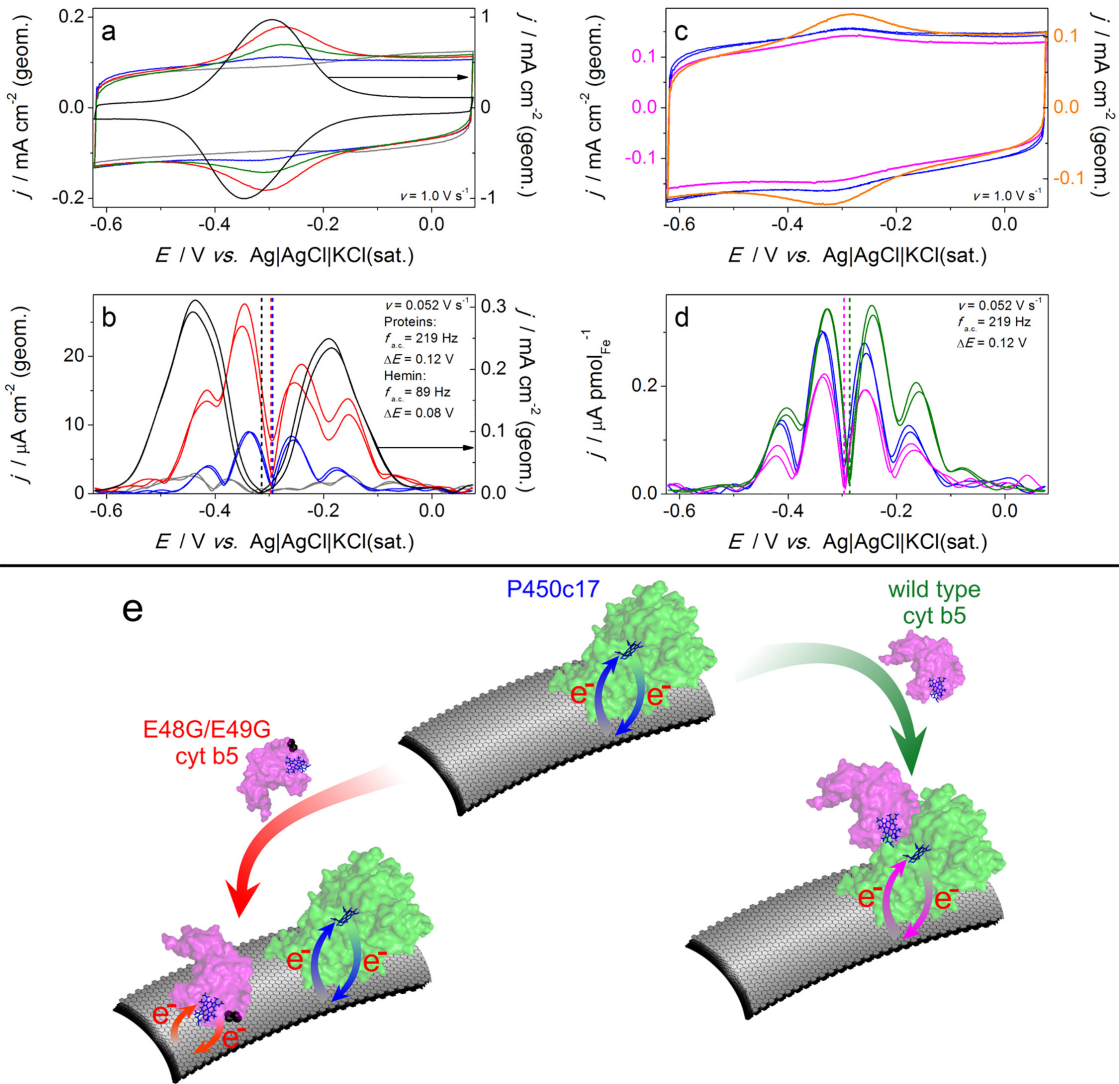
Cyclic voltammetric analysis of P450c17, wt cyt b5, E48G/E49G cyt b5 and sequential additions. **a, c,** d.c. voltammograms and **b, d,** the 6^th^ harmonic of the a.c. voltammograms for the bare (*grey*; left axis) and modified CNT electrodes. **a**, **b,** Adsorption of hemin (black, right axes) produced a much larger faradaic current and an $E_{ac}^{0}$ value (dashed lines) that were at least 0.02 V more negative than those derived from adsorption of proteins (left axes): wt cyt b5 (green), E48G/E49G cyt b5 (red) or P450c17 (blue). **c,** Adsorption of E48G/E49G cyt b5 on P450c17/CNT (blue) produced a substantial increase in the surface concentration of hemin (orange), but negligible changes in *Γ* were derived from adsorption of wt cyt b5 (magenta) on P450c17/CNT. **d,** The faradaic current in the a.c. components for P450c17 (blue) was suppressed upon interaction with wt cyt b5 (magenta); the a.c. data for wt cyt b5 (green) is shown as a control. (**e**) Schematic representation of the modes of interaction of the wt cyt b5 and the E48G/E49G cyt b5 with the P450c17/CNT electrode. All currents were normalized to the geometric electrode surface area (a-c) or to the amount of electroactive heme (d); The electrolyte solution in each case was a deoxygenated aqueous 0.20 M NaCl+0.02 M (K_2_HPO_4_+KH_2_PO_4_), pH = 7.0.

Direct electrochemistry of the proteins could not be achieved efficiently on a supported lipid bilayer that is effectively an insulator. Biomembrane mimics, including polyions [36], could also not be used because they ‘trap’ rather than provide a physiological comparison of protein redox potentials [37]. Therefore, multiwalled carbon nanotube (CNT) based electrodes containing a highly hydrophobic surface, with minimal imperfections [19, 20, 38] were used as a platform for immobilization of proteins (page S11 in S1 File). Direct current (d.c.) and Fourier transformed (FT) alternating current (a.c.) [39, 40] voltammetric techniques were employed to establish the surface concentration of the electrochemically active iron heme and associated kinetic and thermodynamic parameters of the electron transfer, respectively. Fig 3 (blue data) shows the d.c. and 6^th^ harmonic components of a.c. cyclic voltammograms for the CNT electrodes modified with P450c17 solution. Surface concentrations of the redox active heme in the P450c17-modified electrodes were estimated as *ca* 0.4–0.7 ${\text{pmol cm}}_{\text{CNT}}^{-2}$.

In order to be able to identify faradaic response arising from loss of the heme moiety from either the P450c17 or the cyt b5 proteins in our experiments, we initially studied the voltammetry of free hemin adsorbed on CNT-based electrodes. Free hemin showed a well-defined faradaic response (Fig 3A and 3B, black traces) with a charge that corresponded to a surface coverage (*Γ*) of *ca* 30–50 ${\text{pmol cm}}_{\text{CNT}}^{-2}$ representing 0.27–0.45 monolayer coverage. These results were similar to those reported by Sagara *et al*. [41] using highly oriented pyrolytic graphite. The reversible potentials ($E_{ac}^{0}$) derived from the a.c. voltammetric data obtained with P450c17 and hemin-modified CNT electrodes are separated by more than 0.02 V (Table 1) confirming that the surface confined species produced by adsorption of the P450c17 enzyme and the free heme on the CNTs are not the same.

**Table 1:** Reversible potentials ($E_{ac}^{0}$) of the surface confined Fe^3+/2+^ redox couple derived from the a.c. voltammograms (*f* = 219 Hz)*^a^* obtained from CNT electrodes modified with hemin, wt cyt b5, E48G/E49G cyt b5, P450c17, and their combinations.

Adsorbed Species	$E_{ac}^{0}$ / V *vs*. Ag|AgCl|KCl(sat.) ^*b*^
hemin	-0.318
wt cyt b5	-0.285
hemin / wt cyt b5 ^*c*^	-0.319
E48G/E49G cyt b5	-0.295
P450c17	-0.296
wt cyt b5 / P450c17 ^*d*^	-0.296
E48G/E49G cyt b5 / P450c17 ^*d*^	-0.295

^*a*^ Average of the potentials of the central minimum in the 6^th^ a.c. harmonic (envelope presentation, in Fig 3) from the forward and backward d.c. potential sweep directions.

^*b*^ Values are reproducible to ±0.001 V.

^*c*^ Hemin adsorbed on the electrode pre-modified with wt cyt b5.

^*d*^ either wt or E48G/E49G cyt b5 adsorbed on the electrode pre-modified with P450c17.

Table 1 summarizes $E_{ac}^{0}$ data for the individually adsorbed P450c17, wt cyt b5 or E48G/E49G cyt b5 proteins and hemin. The $E_{ac}^{0}$ values were reproducible to ±0.001 V and an experimentally distinguishable 0.01 V difference was evident between the wt cyt b5 and the E48G/E49G cyt b5. Importantly, deposition of P450c17, wt cyt b5 or E48G/E49G cyt b5 on the CNT-based electrodes all produced a surface-confined species, virtually devoid of signals arising from the loss of heme (Fig 3A and 3B, Table 1). Analysis of the experimental a.c. voltammograms indicated some heterogeneity of these modified electrodes as expected [42]. Further discussion of the modes of adsorption of the proteins on the CNT surface is provided in the Supplementary Information (Figures I-K, Table C and accompanying discussion on pages S14-S15 in S1 File).

$E_{ac}^{0}$ values discussed above were compared with those found upon addition of wt or E48G/E49G cyt b5 proteins to CNTs pre-modified with P450c17 (Table 1 and Fig 3C and 3D). Significantly, these voltammetric data show that adsorption of the wt cyt b5 did not change or just slightly decreased (by 0–5%) the surface concentration, *Γ*, of the electrochemically active heme (Fig 3C magenta trace). This contrasted with the pronounced enhancement in *Γ* upon modification of the P450c17/CNT electrode assembly with E48G/E49G cyt b5 (Fig 3C orange trace). Thus, the voltammetric data indicated that the E48G/E49G cyt b5 was immobilized on the CNT surface with no specific interaction with the P450c17. These results are in good agreement with the QCM studies using a lipid layer.

To further explore the nature of the interaction between wt cyt b5 and the P450c17-modified electrodes we used simultaneous analysis of both d.c. (Fig 3C) and a.c. voltammetric data (Fig 3D). While the d.c. component showed no or very small decrease in protein coverage, *Γ* (Fig 3C, wt cyt b5/P450c17/CNT, magenta trace *vs*. P450c17/CNT alone, blue trace), the a.c. voltammograms exhibited an accompanying significant suppression of the harmonic components by 35 ± 6% reflecting a deceleration of the electron transfer kinetics (Fig 3D, wt cyt b5/P450c17/CNT, magenta trace *vs*. P450c17/CNT alone, blue trace). The shape of the a.c. faradaic signal is highly sensitive to the nature of the redox process, and is expected to change substantially when mixed adsorbates with different signatures are introduced. Importantly, this was not found when wt cyt b5 was added to P450c17/CNT. Furthermore, no positive shift of the reversible potential was observed as would be expected if cyt b5 was adsorbed directly on the CNTs (see Table 1; Fig 3D, green trace). Thus, the FT a.c. voltammetry observations support preferential deposition of ‘electrochemically silent’ wt cyt b5 on the surface of electroactive P450c17, in confirmation of negligible changes in the d.c. faradaic charge. Taken together, the a.c. and d.c. voltammetry data suggest that the effect of wt cyt b5 addition on a P450c17-CNT electrode is the modulation of the charge transfer kinetics associated with P450c17 protein complex(es) as illustrated in Fig 3E. The significant differences in the adsorption of the wild type and E48G/E49G cyt b5 proteins on the P450c17-modified electrodes also indicated that the P450c17 enzyme experienced no structural damage upon adsorption on the CNT (see Fig 3C).

## Discussion

Despite the decade long knowledge that cyt b5 plays a significant role in the 17,20-lyase reaction of P450c17 and hence androgen synthesis, the nature of this interaction and regulatory mechanism has not been identified. We have approached this problem using a number of complementary *in silico*, *in vivo* and *in vitro* techniques in order to provide a comprehensive biophysical analysis.

The FRET analyses in living cells showed a heterodimeric interaction between P450c17 and cyt b5 and were conducted [22], but not reported previously, as part of an examination of steroidogenic P450 enzyme interactions. It is known that proteins can form nonspecific aggregates at high expression levels [43] but the lack of heterodimerization between P450c17 and P450arom, as shown previously [22], argues strongly that the FRET complexes detected here for P450c17 and cyt b5 result from highly specific interactions. Furthermore, the FRET complexes observed between P450c17 and CPR were as expected from functional studies. Importantly, the FRET level of P450c17 with cyt b5 was much greater than even with CPR, clearly indicating that a close and specific binding of cyt b5 and P450c17 occurs in live cells.

Computational docking predicted the binding interface and key interactions. The predicted major contributors to the binding energy are polar interactions between cyt b5 residues Glu48, Glu49, Glu42 and Asp65 to P450c17 residues Ser427, Arg358, Arg347 and Lys88, respectively. The good agreement with the recent cross-linking studies by Peng *et al*. [4] provided confidence in our model. Importantly, the modeling also predicted that E48G/E49G cyt b5 would not form an energetically favorable association with P450c17.

QCM analyses were conducted using a composite DMPC-cholesterol lipid bilayer to mimic protein interactions as might occur *in vivo*. Our earlier studies using P450c17 showed the specific interactions between P450c17 with CPR [22] but were not extended to cyt b5. NMR studies probing the interaction between P450c17 and cyt b5 have been conducted in solution [15, 35]. These experiments were conducted using a soluble, carboxy-terminally truncated form of cyt b5 that has only 1/6^th^ of the capacity to stimulate 17,20-lyase activity of P450c17 [44]. Hence protein interactions observed previously might not have accurately reflected those that occur *in vivo*. Thus, we compared the protein-protein interactions between the wt cyt b5 or E48G/E49G cyt b5 with P450c17 + CPR complexes on a biomimetic membrane using a QCM. In the case of wt cyt b5, the protein deposition fingerprint (Δ*f*-*t*, Δ*D*-*t* and Δ*f*-Δ*D* in Fig 2) indicated that the wt cyt b5 specifically bound to P450c17 + CPR complexes formed in the initial solution and therefore co-deposited into the membrane modified QCM sensor. In contrast, the E48G/E49G cyt b5 deposited more rapidly and non-specifically to the membrane and co-deposited competitively with P450c17 + CPR. Further evidence was gleaned with the concentration-dependent binding for the E48G/E49G cyt b5 deposition. Thus, the QCM data support the concept that wt cyt b5 interacts specifically with the P450c17 in the membrane and furthermore agrees well with our *in silico* model. Importantly, the QCM data highlighted the crucial importance of an electrostatic network within the interfacial domains between wt cyt b5 with P450c17 and specifically the glutamic acid residues 48 and 49 on the cyt b5 for protein-protein interactions in a membrane.

Direct electrochemical studies specifically enabled us to demonstrate that adsorption of the wt cyt b5 on the P450c17/CNT composite decreased the rate of the electron transfer between P450c17 and the electrode, although the wt cyt b5 itself was electrochemically inactive. This effect is consistent with wt cyt b5 acting allosterically on P450c17. In contrast, E48G/E49G cyt b5 adsorbed non-specifically and did not have an effect on P450c17 electrochemistry, supporting the claim that the effect of wt cyt b5 on P450c17 was specific. Thus, these electrochemical experiments illustrate modulation of the electron transfer properties of P450c17 with and without cyt b5 and illustrate that this direct interaction requires the E48 and E49 residues.

The slower electron transfer kinetics for the P450c17/CNT electrodes upon adsorption of wt cyt b5 (Fig 3D) is most likely of ‘biological’ origin, *i*.*e*. reflects the kinetic effect of protein-protein interaction on the rate of electron transfer in (and out of) the heme moiety (Fig 3E). Alternative ‘physicochemical’ origins, due to surface effects of the electrode are less likely. In principle, the magnitude of the a.c. current might be modulated due to changes in heterogeneity of the surface-confined protein upon adsorption of foreign species. However, the voltammetric data were reproducible in terms of (i) negligible changes in the faradaic d.c. charge (0–5%), (ii) the decrease in the faradaic a.c. current (35 ± 6%), (iii) maintaining the shape of harmonics, and (iv) no change in $E_{ac}^{0}$. Physicochemical effects are typically more erratic in nature and do not explain these data. Thus, we attribute the role of cyt b5 as regulating the rate of electron movement for P450c17.

It has been long suggested that the interaction of P450c17 and cyt b5 induces conformational changes in P450c17 as part of its mechanism of action [5]. Our electrochemistry data provides the first functional evidence to support the suggestion that changes in the conformation of P450c17 account for the slowing in the rate of electron transfer between P450c17 and the electrode or another molecule.

In summary, our comprehensive examination of the interaction between P450c17 with CPR and either wt cyt b5 or a functionally defective mutant E48G/E49G cyt b5 employed four biophysical approaches. FRET analysis was used to examine interactions within living cells and *in silico* modeling identified the sites of interaction likely to be disrupted between the E48G/E49G cyt b5 and P450c17. QCM identified specific protein-protein interactions in a biomimetic lipid membrane and combined d.c. and a.c. voltammetric analysis revealed that the wt cyt b5, but not E48G/E49G cyt b5, altered the kinetics of electron transfer between the electrode and the P450c17. This hitherto unknown effect of cyt b5 on the electron transfer kinetics of P450c17 provides novel mechanistic insight into how cyt b5 regulates the 17,20 lyase activity of P450c17.

## Supporting Information

S1 FileSupporting Information.pdf, contains: Table A: Output energies for the top 10 poses from RosettaDock; Figure A: TEM images of CNTs; Figure B: Western immunoblot of cyt b5; Figure C: Activity of P450c17-eCFP and P450c17-eYFP using pregnenolone as the substrate; Figure D: Photographs of the CNT suspension and CNT-modified GC electrode; Figure E: Stereo images of the P450c17 (green) and cyt b5 (magenta) model complex in Fig 1B; Figure F: Mutated residues of cyt b5; Figure G: Arg449 position in the protein-protein interface; Table S2B: Percentage occupancy of hydrogen bonds between the interface of P450c17 and cyt b5 during the molecular dynamics simulations; Figure H: QCM data; Supplementary discussions: Choosing an electrode platform for probing the P450c17 and cyt b5 electrochemistry; Figure I: Electrocatalytic properties of P450c17 adsorbed on CNT; Figure J: Oxygen electroreduction catalyzed by protein- and hemin-modified CNT electrodes; Supplementary discussions: Enzymatic capacity of P450c17 immobilized on a CNT-based electrode; Supplementary discussions: Adsorption of cyt b5 on bare CNT-based electrodes; Figure K: Electrochemistry of cyt b5-polymyxin B films; Table C: Reversible potentials ($E_{\text{ac}}^{0}$) for PGE electrodes modified with cyt b5 with polymyxin B films; List of abbreviations; Supplementary references.(PDF)
