# Supplementary material for: Mechanistic Scrutiny Identifies a Kinetic Role for Cytochrome b5 Regulation of Human Cytochrome P450c17 (CYP17A1, P450 17A1)
Source: PLoS One. 2015 Nov 20;10(11):e0141252. doi: 10.1371/journal.pone.0141252 (PMC4654539; doi:10.1371/journal.pone.0141252)
Supplement: S1 File — (PDF) [file pone.0141252.s001.pdf]

S1 Supporting Information

Mechanistic scrutiny identifies a kinetic role for cytochrome b5 regulation of human cytochrome P450c17 (CYP17A1, P450 17A1)

Alexandr N. Simonov,[1] Jessica K. Holien,[2] Joyee Chun In Yeung,[1] Ann D. Nguyen,[3] C. Jo Corbin,[3] Jie Zheng,[4] Vladimir L. Kuznetsov,[5] Richard J. Auchus,[6] Alan J. Conley,[3] Alan M. Bond,[1] Michael W. Parker,[2,7] Raymond J. Rodgers,[8] Lisandra L. Martin[1*]

1 School of Chemistry, Monash University, Clayton, Victoria 3800, Australia, **2** ACRF Rational Drug Discovery Centre, St. Vincent's Institute of Medical Research, Fitzroy, Victoria 3065, Australia, **3** School of Veterinary Medicine, University of California, Davis, California 95616, USA, **4** Department of Physiology and Membrane Biology, School of Medicine, University of California, Davis, California 95616, USA, **5** Boreskov Institute of Catalysis, Prospekt Lavrentieva 5, Novosibirsk, 630090, Russia, **6** Division of Metabolism, Endocrinology and Diabetes, Department of Internal Medicine, University of Michigan, Ann Arbor, Michigan 48109, USA, **7** Department of Biochemistry and Molecular Biology, Bio21 Molecular Science and Biotechnology Institute, University of Melbourne, Parkville, Victoria 3010, Australia, **8** Discipline of Obstetrics and Gynaecology, School of Paediatrics and Reproductive Health, Robinson Research Institute, University of Adelaide, Adelaide, South Australia 5005, Australia

Supporting Information Table of Contents

	Page
Table A | Output energies for the top 10 poses from RosettaDock	S3
Figure A | TEM images of CNTs	S4
Figure B | Western immunoblot of cyt b5	S5
Figure C | Activity of P450c17-eCFP and P450c17-eYFP using pregnenolone as the substrate	S5
Figure D | Photographs of the CNT suspension and CNT-modified GC electrode	S6
Figure E | Stereo images of the P450c17 (green) and cyt b5 (magenta) model complex in Figure 1b.	S7
Figure F | Mutated residues of cyt b5	S8
Figure G l Arg449 position in the protein-protein interface	S9
Table B | Percentage occupancy of hydrogen bonds between the interface of P450c17 and cyt b5 during the molecular dynamics simulations	S10
Figure H l QCM data	S11
Choosing an electrode platform for probing the P450c17 and cyt b5 electrochemistry	S12
Figure I | Electrocatalytic properties of P450c17 adsorbed on CNT	S13
Figure J | Oxygen electroreduction catalyzed by protein- and hemin-modified CNT electrodes	S14
Enzymatic capacity of P450c17 immobilized on a CNT-based electrode	S15
Adsorption of cyt b5 on bare CNT-based electrodes	S16
Figure K | Electrochemistry of cyt b5-polymyxin B films	S17
Table C | Reversible potentials (E^0_{ac}) for PGE electrodes modified with cyt b5 with polymyxin B films	S18
List of abbreviations	S22
Supplementary references	S23

Table A | Docking Score output from RosettaDock for the top 10 poses. The score is a direct correlation to the energy.

Docking Pose	Total Score / kcal	RMSD from initial pose	Interface Score* / kcal
**1**	-512.291	5.18	-5.866
**2**	-512.843	4.121	-5.581
**3**	-512.369	4.145	-5.542
**4**	-512.291	5.392	-5.407
**5**	512.004	3.068	-5.334
**6**	-511.455	5.14	-5.221
**7**	-511.86	5.365	-5.072
**8**	-511.717	5.036	-5.031
**9**	-511.274	24.319	-4.77
**10**	-511.591	6.491	-4.705

*The interface score is the energy of the whole complex minus the energy of each individual protein.

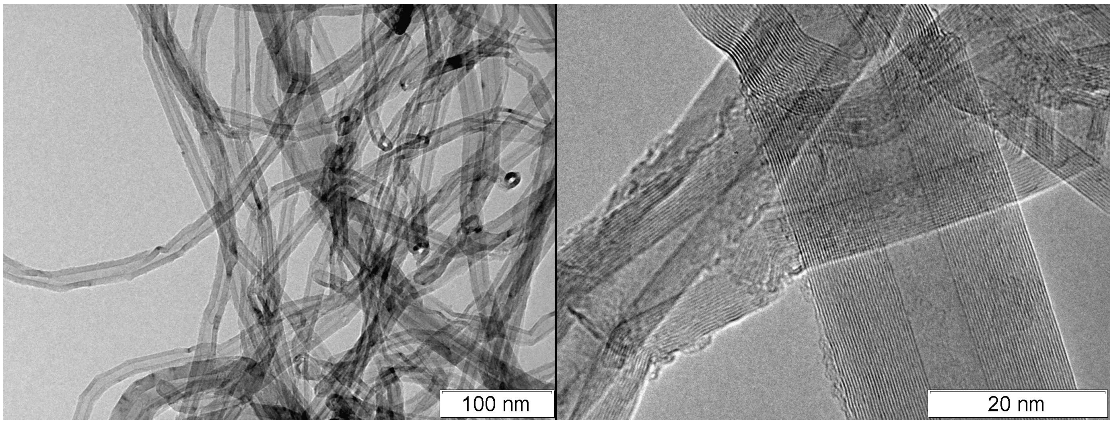

Figure A | TEM images of the multiwall carbon nanotubes used for fabrication of the electrodes.

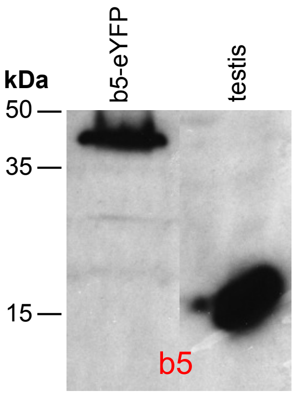

Figure B | Western immunoblot of cyt b5.

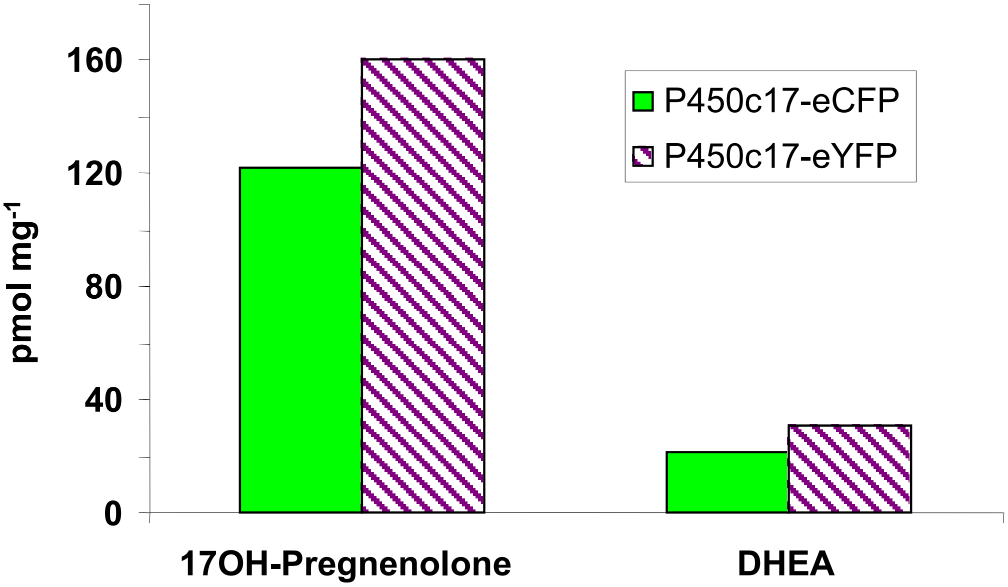

Figure C | Metabolism of pregnenolone by COS1 cells transiently transfected with P450c17 fusion constructs. Both 17α-hydroxylase and 17,20-lyase activity were retained by P450c17-eCFP and P450c17-eYFP fusion proteins, as determined by formation of 17OH-pregnenolone and DHEA.

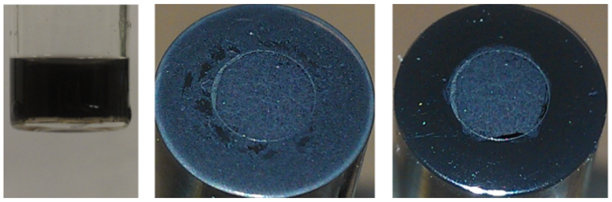

Figure D | Photographs of 0.2 mg ml^{-1} suspension of CNTs in isopropanol (left), CNT layer deposited on the whole surface of the electrode (middle) and the same layer after removal of the CNTs deposited on the isolating sheath (right).

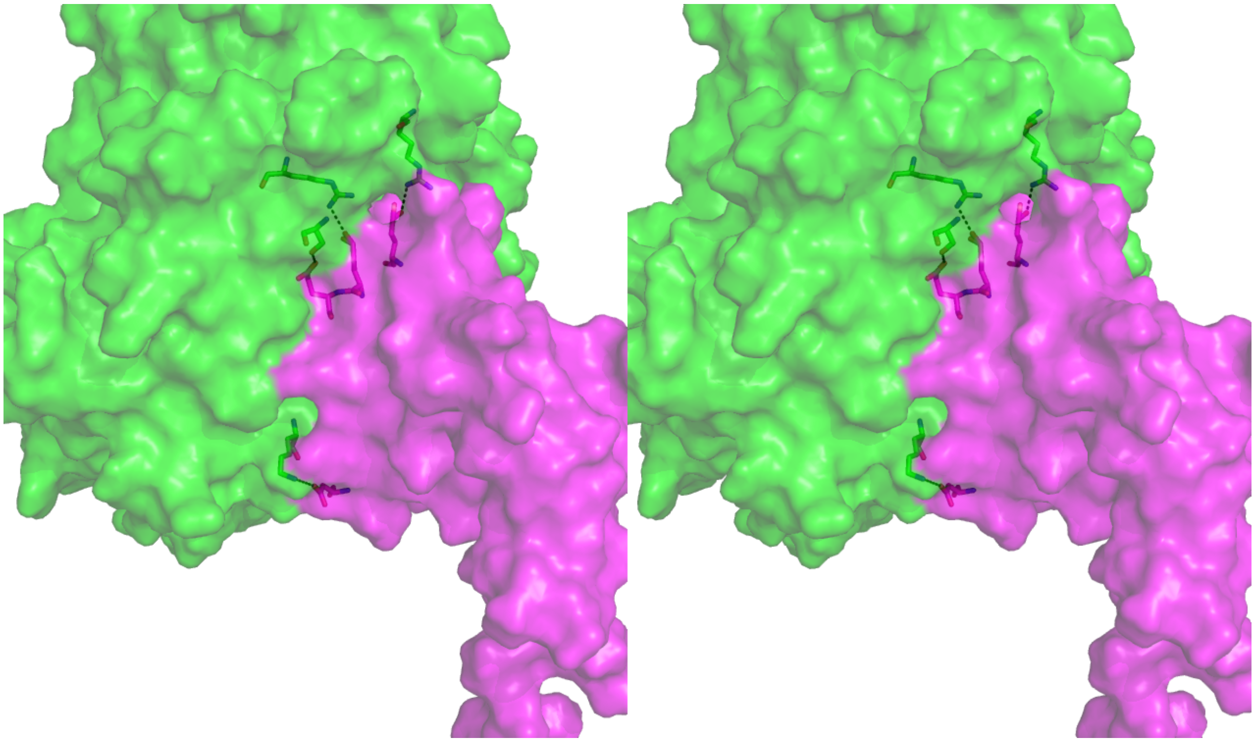

Figure E | Cross-eyed stereo representation of Figure 1b. Model of the complex between P450c17 (green) and cyt b5 (magenta) shown in rendered style. Polar interactions are shown as black dashed lines. Those residues involved in polar interactions are highlighted via sticks. The labels are shown in Figure 1b.

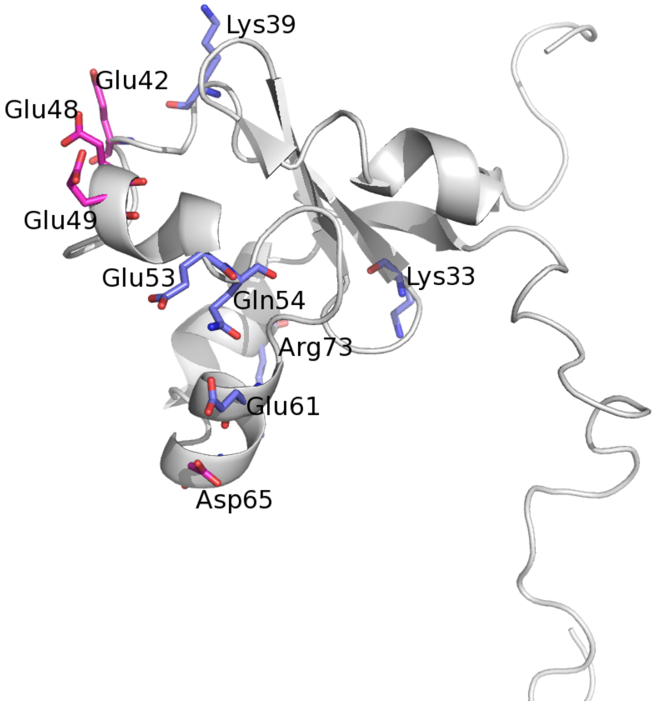

Figure F | Mutated residues of cyt b5. Shown in purple sticks are residues of cyt b5 that when mutated displayed no change to wild type 17,20-lyase activity S(1). Notably, none of these residues are near the putative key residues in the predicted sites of protein-protein interaction (shown as magenta sticks).

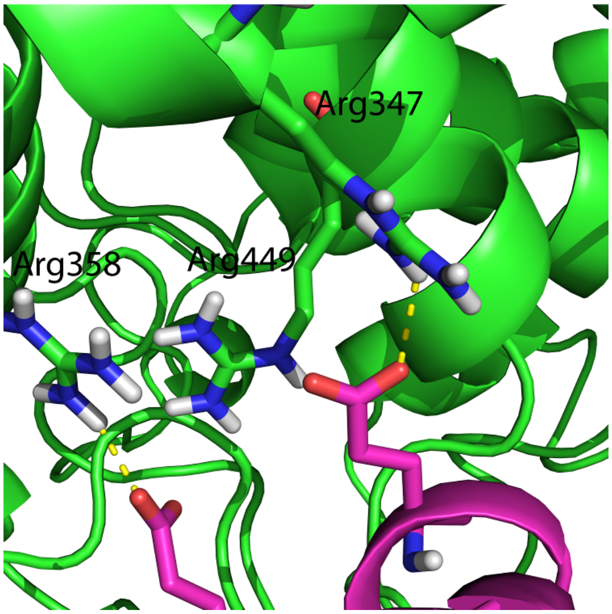

Figure G | Arg449 position in the protein-protein interface. Although in this static dock Arg449 is not directly involved in a polar interaction, its side-chain is free to move, and in a fluid protein environment this residue would be expected to play a role in stabilizing the interface.

Table B | Percentage occupancy of hydrogen bonds between the interface of P450c17 and cyt b5 during the molecular dynamics simulations. A hydrogen bond was counted as within a donor-acceptor distance within 3Å and angle limits of 20°.

P450c17	Cyt b5	Occupancy*
Arg347	Glu42	120.69%
Arg358	Glu48	96.52%
Ser427	Glu49	63.82%
Arg449	Glu48	61.91%
Lys89	Asp65	27.01%
Lys88	Asp65	14.02%
Arg449	Glu42	9.17%
Lys88	Glu61	6.52%

*Occupancy can be greater than 100% as a given residue pair may contain more than one hydrogen bond, each of which was counted separately.

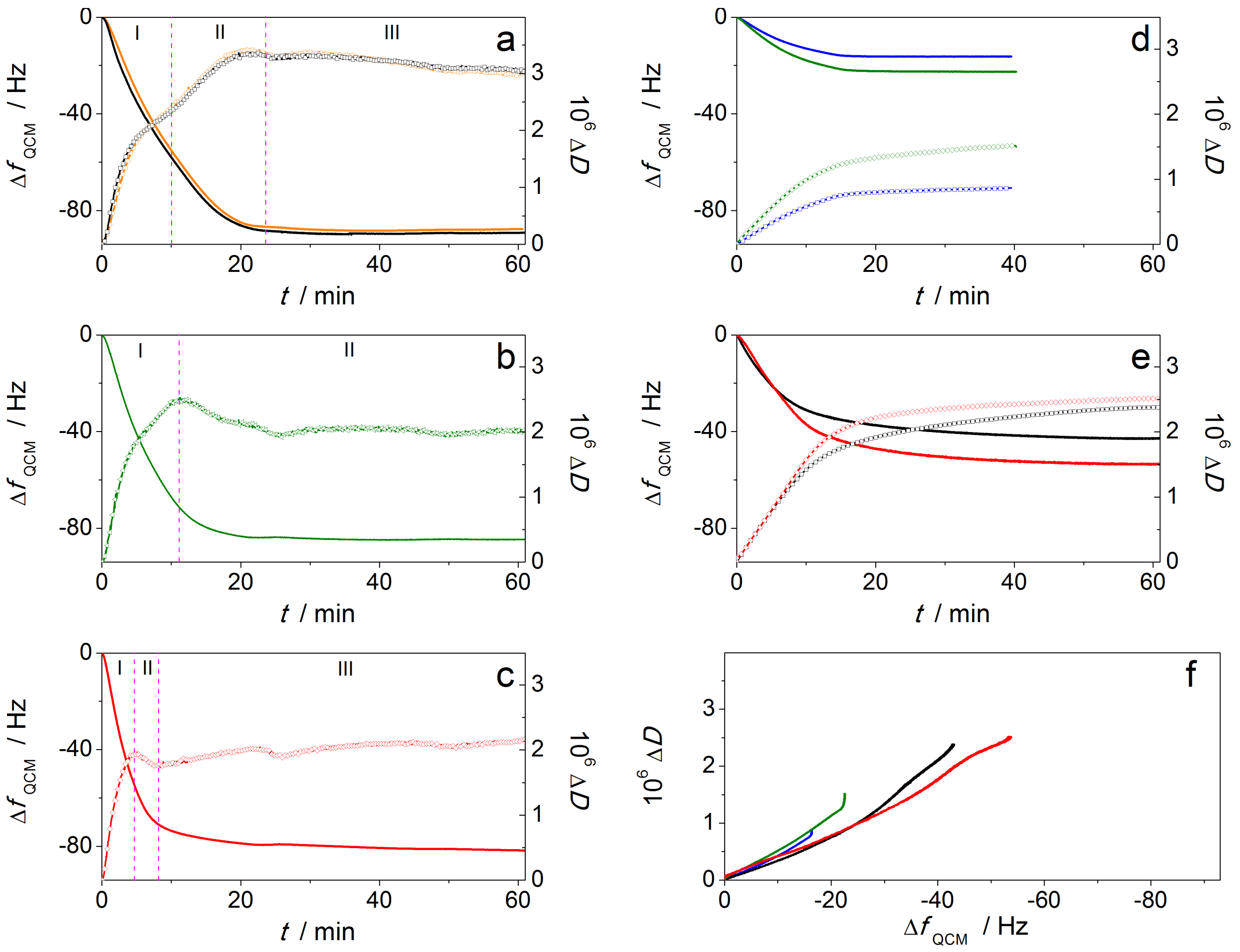

Figure H | QCM data. (**a-e**) QCM data for the temporal changes in frequency (lines, left axes) and dissipation (symbols + lines, right axes) (7th harmonic; Δf_{QCM} data are normalized to the overtone number) derived from deposition of protein solutions containing (**a**) 20 nM P450c17 + 20 nM CPR (orange), 20 nM P450c17 + 20 nM CPR+100 nM wt cyt b5 (black); (**b**) and (**c**), 20 nM P450c17 + 20 nM CPR + 20 (green) or 100 nM E48G/E49G cyt b5 (red); (**d**), 20 nM wt cyt b5 (blue), 20 nM E48G/E49G cyt b5 (green); (**e**), 100 nM wt cyt b5 (black), 100 nM E48G/E49G cyt b5 (red). The vertical dashed lines divide these kinetic data into characteristic deposition stages labeled with roman numerals (I – III) as defined in Fig. 2b (main text). (**f**) Δf_{QCM} *vs.* ΔD plots constructed from data in panels (d) and (e). All solutions contained proteins in 0.02 M NaCl + 0.02 M (K_2HPO_4 + KH_2PO_4) (pH = 7.0), and were introduced onto a composite DMPC-cholesterol (20 mol.%) layer, predeposited on a mpa layer adhered to a gold-coated quartz crystal (*ca* 1 cm diameter) at 295 K and with a flow rate of 0.05 ml min^{-1}. Note: the wider Δf_{QCM} scale was used in panel f in order to compare these data with those in Fig. 3d.

The electrode platform for probing the P450c17 and cyt b5 electrochemistry

Choosing an appropriate electrode platform for studying the electrochemistry of proteins is crucial for obtaining reliable and interpretable data. Even the simplest proteins offer challenges for voltammetric studies, especially when denaturation of biomolecules occurs during redox reactions or even simply upon adsorption on an unnatural surface. For the analysis of specific interactions between P450c17 and cyt b5 a simple electrode configuration based on the natural affinity of P450c17 to a hydrophobic membrane environment was used. Carbon-based electrodes are often effective interfaces for protein voltammetry, yet the denaturation of proteins with carbon electrodes is attributed to impurities (amorphous carbon deposits, metals and metal oxides, etc.) and surface irregularity (defects) disrupting the tertiary protein structure. Indications of this have been obtained in our preliminary electrochemical studies on P450c17 with the use of bare glassy carbon and PGE electrodes, but much less with the PGB platform. Taking all of the above into account, a carbon material virtually devoid of the mentioned imperfections, namely, a network of multiwall carbon nanotubes subjected to extreme thermal treatment (2800°C) under high purity Ar atmosphere (hereinafter, CNTs) S(2, 3) was chosen as a support for our voltammetric experiments. Since the CNT surface is dominated by highly hydrophobic graphite basal plane with minimal amount of imperfections of any kind, CNTs might be expected to be favourable for adsorption of intact P450c17. Indeed, as shown in Fig. 3 (main text) and discussed in the paper, near-ideal surface of CNTs provided efficient adsorption of P450c17 without any significant loss of heme from the protein. As expected, these P450c17-modified CNT electrodes exhibited catalytic activity for oxygen reduction, as did the hemin-modified ones (Figs. D and E), with the formation of hydrogen peroxide as a dominant product S(4, 5). This was discerned by rapid loss of the faradaic signals from the surface-confined iron heme and also analysis of the products formed during controlled potential electrolysis of the air-saturated aqueous pregnenolone solutions for the composite P450c17/CNT electrode (see section 'Enzymatic capacity of P450c17 immobilized on a CNT-based electrode' on page S9). The lack of efficiency and selectivity of hydroxylation of pregnenolone using the P450c17-modified CNT electrodes is not unexpected, as the natural enzymatic generation of 17α-hydroxy-pregnenolone most probably requires complexation of P450c17 with CPR S(6),S(7).

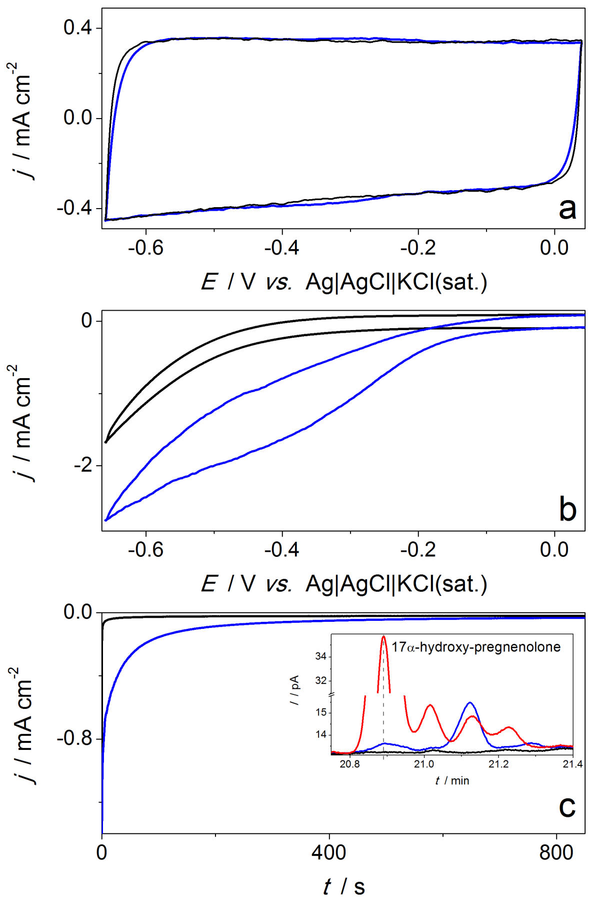

Figure I | Electrocatalytic properties of P450c17 adsorbed on CNT. D.c. voltammograms [v = 0.40 (a) and 0.10 V s^{-1} (b)] for a CNT – carbon cloth composite electrode modified with P450c17 before (*blue*) and after (*black*) chronoamperometric catalytic testing with (**a**) deoxygenated and (**b**) air-saturated 3 mM pregnenolone + 0.20 M NaCl + 0.02 M (K_2HPO_4 + KH_2PO_4) aqueous electrolyte solution (pH = 7.0). Panel (**c**) shows two consecutive chronoamperograms (*blue* – first measurement; *black* – second measurement) obtained at -0.31 V vs. Ag|AgCl|KCl (sat.) during 1200 s with a P450c17/CNT/carbon cloth composite electrode in the presence of pregnenolone in the air-saturated electrolyte solution. Inset in panel (**c**) shows fragments of the gas chromatograms (flame-ionization detector) for the standard solution of 17α-hydroxy-pregnenolone (*red*), initial unelectrolyzed (*black*) and electrolyzed using a P450c17/CNT/carbon cloth composite electrode (*blue*) 3 mM pregnenolone + 0.20 M NaCl + 0.02 M (K_2HPO_4 + KH_2PO_4) solution. Currents are normalized to the geometric surface area of the electrode.

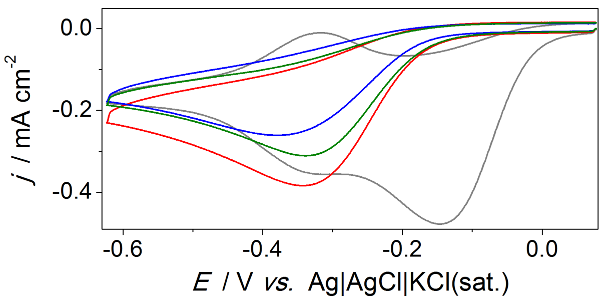

Figure J | Oxygen electroreduction catalyzed by protein- and hemin-modified CNT electrodes. D.c. voltammograms (v = 0.10 V s^{-1}) for CNT electrodes modified with hemin (*grey*), wt cyt b5 (*green*), E48G/E49G mutant cyt b5 (*red*) and sequentially with P450c17 and wt cyt b5 (*blue*) in contact with air-saturated 0.20 M NaCl + 0.02 M (K_2HPO_4 + KH_2PO_4) aqueous electrolyte solution (pH = 7.0). Higher electrocatalytic activity of the hemin-modified electrode is due to a two orders of magnitude higher concentration of the $Fe^{3+/2+}$ species as compared to the protein-based electrodes. Currents are normalized to the geometric surface area of the electrode.

Enzymatic capacity of P450c17 immobilized on a CNT-based electrode

Controlled potential electrolysis of the air-saturated aqueous 3 mM pregnenolone solution (0.20 M NaCl + 0.02 M (K_2HPO_4 + KH_2PO_4)) was undertaken with the use of a composite electrode fabricated *via* adsorption of P450c17 on CNTs immobilized on a carbon cloth to test the enzymatic capacity of these P450c17-modified CNT-electrodes. Reductive currents monitored at the constant potential of -0.31 V, where O_2 electroreduction was predominantly catalyzed by the iron heme rather than by a carbon support, degraded to the values obtained with a protein-free electrode within 10 min under the employed electrolysis conditions (Fig. I). Complete degradation of the iron heme catalytic species was confirmed by chronoamperometric and voltammetric measurements in air-saturated and deoxygenated solutions. Gas chromatograms (flame ionization detector) of the trimethylsilylated dichloromethane or methyl *tert*-butyl ether extracts from the electrolyzed pregnenolone solutions exhibited a peak at the retention time of trimethylsilylated 17α-hydroxy-pregnenolone as well as other peaks of unknown origin at slightly higher retention times, none of which were found in chromatograms obtained with the initial non-electrolyzed pregnenolone solution (Fig. I, panel c). The concentration of 17α-hydroxy-pregnenolone was below 10^{-9} M, while the charge passed during the electrolysis provided two-electron reduction of $1.8 \cdot 10^{-5}$ M O_2 predominantly to H_2O_2. Most probably, hydrogen peroxide can assist enzymatic hydroxylation of pregnenolone by P450c17 as well as can chemically oxidize the steroid hormone substrate S(4-6, 8). Obviously, the latter pathway lacks regioselectivity and can result in the formation of the unidentified compounds, which were detected chromatographically in the electrolyzed pregnenolone solution as peaks in the vicinity of the trimethylsilylated 17α-hydroxy-pregnenolone signal. The ability to detect 17α-hydroxy-pregnenolone in solutions electrolyzed using P450c17/CNT electrodes provide confidence that immobilization of P450c17 is functionally competent and catalytically active during the electrochemical experiments.

Adsorption of cyt b5 on bare CNT-based electrodes

Although the cyt b5 contains an abundance of negatively charged residues (as confirmed by voltammetric studies on cyt b5-polymyxin B complexes immobilized on PGE electrodes shown in Fig. K), adsorption of both wild type and E48G/E49G mutant cyt b5 proteins on CNT electrodes produced pronounced voltammetric signals from the surface confined iron heme (Fig. 2 of the main text). Typically, the surface concentration of the Fe-containing species on the CNT electrodes obtained with E48G/E49G cyt b5 was higher than observed after adsorption of wt cyt b5, as might be expected when the surface-exposed glutamic acid residues were replaced with glycine. Moreover, the Γ values attained upon modification of a CNT electrode with cyt b5 were *ca* 2-3 times higher than those measured with P450c17 electrode.

The mechanism of adsorption of cyt b5 on CNT electrodes is unknown. The apparent reversible potentials for the $Fe^{3+/2+}$ process derived from the a.c. voltammetric analysis of CNT electrodes modified with wt and E48G/E49G cyt b5 differ from each other and, most importantly, from E^0_{ac} for pure hemin (Table 1 in the main text). Furthermore, these differences in E^0_{ac} follow the same tendency when wild type and mutant proteins were adsorbed on pure CNTs or connected to a PGE electrode *via* polymyxin B, *i.e.* under conditions where denaturation was less likely (*cf.* data in Table 1 in the main text and Table C). Substantial difference in E^0_{ac} obtained with cyt b5-modified CNT and polymyxin B/PGE electrodes can be explained by the formation of complexes between polymyxin B and proteins, presumably, *via* the carboxylic functional groups of the iron heme S(9). Alternatively, CNT electrodes modified with cyt b5 exhibit pronounced catalytic activity for oxygen reduction (Fig. J), notwithstanding the coordination of the heme iron atom to two histidine residues, that would preclude access of molecular oxygen S(10). A similar effect was reported by Zhang *et al.* S(11) when cytochrome c_3 was adsorbed on a glassy carbon electrode. Thus, the cyt b5 proteins could be partially disrupted upon adsorption on CNTs in order to provide access of molecular oxygen to the iron atom of the heme moiety. However, there are no circumstances we can envisage that affects the major conclusions of the study on interactions between P450c17 and wild type or E48G/E49G cyt b5 proteins.

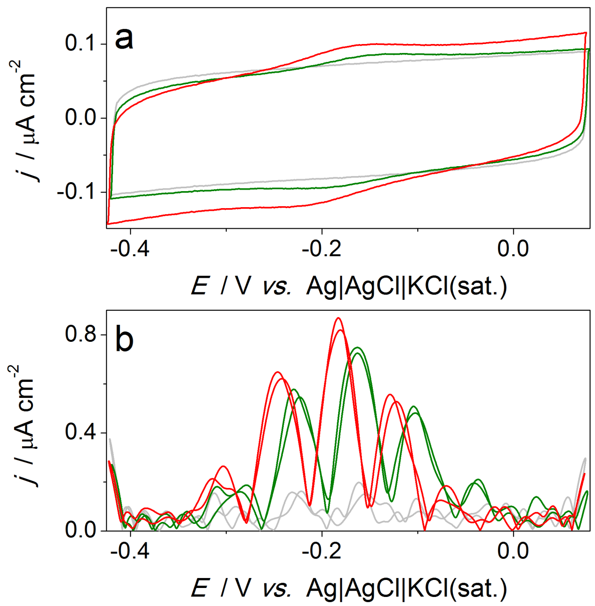

Figure K | Voltammetry of cyt b5–polymyxin B films. (**a**) D.c. voltammograms ($v = 1.0$ V s^{-1}) and (**b**) 7th harmonic components of a.c. voltammograms (f_{ac} = 9.02 Hz; $\Delta E = 0.12$ V; v = 0.07451 V s^{-1}) for a bare PGE electrode (*grey*) and PGE electrodes modified with polymyxin B containing solutions of wt cyt b5 (*green*) or E48G/E49G mutant cyt b5 (*red*) in contact with deoxygenated 0.5 mg ml^{-1} polymyxin B + 0.20 M NaCl + 0.02 M (K_2HPO_4 + KH_2PO_4) aqueous electrolyte solution (pH = 7.0). Currents were normalized to the geometric surface area of the electrode.

Table C | Reversible potentials (E^0_{ac}) of the surface confined $Fe^{3+/2+}$ redox couple derived from the a.c. voltammograms (f_{ac} = 89 Hz)[a] obtained for PGE electrodes modified with cyt b5-polymyxin B complexes.

**Mutation**	$\boldsymbol{E^0_{ac}}$ **/ V** ***vs.*** **Ag|AgCl|KCl(sat.)** [b]
Wild type	-0.153
E74G	-0.152
R52G	-0.152
E48G/E49G	-0.169

[a] Estimated as the average of potentials of the central minimum in the 6th harmonic component (envelope presentation) from the forward and backward d.c. potential sweep directions. [b] All values were subject to 0.003 V uncertainty. Lower reproducibility as compared to CNT-based electrodes (Table 1 in the main text) was due to enhanced interference of the background signals from PGE electrodes.

List of abbreviations

17-OH-preg	17α-hydroxypregnenolone
a.c.	alternating current
Arg	arginine
Asp	aspartic acid
BSTFA	N,O-bis(trimethylsilyl) trifluoroacetamide
cDNA	complementary deoxyribonucleic acid
CNT	multiwall carbon nanotubes
CPR	NADPH-cytochrome P450 oxido-reductase
cyt b5	cytochrome b5
d.c.	direct current
DHEA	dehydroepiandrostenedione
DMPC	1,2-dimyristoyl-sn-glycero-3-phosphocholine
E48G/E49G cyt b5	double mutant E48G/E49G cytochrome b5 (E = glutamic acid; G = glycine)
eCFP	cyan fluorescent protein
eYFP	yellow fluorescent protein
FRET	fluorescence resonance energy transfer
FT	Fourier transform
GC	glassy carbon
Glu	glutamine
Lys	lysine
mpa	mercaptopropionic acid
NADPH	nicotinamide adenine dinucleotide phosphate
P450	cytochrome P450
P450c17	cytochrome P450 17α-hydroxlase (CYP17A1)
PGB	pyrolytic graphite basal plane
PGE	pyrolytic graphite edge plane
preg	pregnenolone
QCM	quartz crystal microbalance
Ser	serine
TEM	transmission electron spectrocopy
TMCS	trimethylchlorosilane
wt cyt b5	wild type cytochrome b5

Supplementary References

S1. Naffin-Olivos JL & Auchus RJ (2006) Human Cytochrome b5 Requires Residues E48 and E49 to Stimulate the 17,20-Lyase Activity of Cytochrome P450c17†. *Biochemistry* 45(3):755-762.

S2. Elumeeva KV*, et al*. (2013) Reinforcement of CVD grown multi-walled carbon nanotubes by high temperature annealing. *AIP Advances* 3(11):112101.

S3. Cherstiouk OV*, et al*. (2010) Electrocorrosion properties of multiwall carbon nanotubes. *physica status solidi (b)* 247(11-12):2738-2742.

S4. Zu X, Lu Z, Zhang Z, Schenkman JB, & Rusling JF (1999) Electroenzyme-Catalyzed Oxidation of Styrene and cis-β-Methylstyrene Using Thin Films of Cytochrome P450cam and Myoglobin. *Langmuir* 15(21):7372-7377.

S5. Estavillo C, Lu Z, Jansson I, Schenkman JB, & Rusling JF (2003) Epoxidation of styrene by human cyt P450 1A2 by thin film electrolysis and peroxide activation compared to solution reactions. *Biophysical Chemistry* 104(1):291-296.

S6. Krishnan S, Wasalathanthri D, Zhao L, Schenkman JB, & Rusling JF (2011) Efficient Bioelectronic Actuation of the Natural Catalytic Pathway of Human Metabolic Cytochrome P450s. *Journal of the American Chemical Society* 133(5):1459-1465.

S7. Naffin-Olivos JL & Auchus RJ (2005) Human Cytochrome b5 Requires Residues E48 and E49 to Stimulate the 17,20-Lyase Activity of Cytochrome P450c17†. *Biochemistry* 45(3):755-762.

S8. Auchus RJ & Miller WL (1999) Molecular Modeling of Human P450c17 (17α-Hydroxylase/ 17,20-Lyase): Insights into Reaction Mechanisms and Effects of Mutations. *Molecular Endocrinology* 13(7):1169-1182.

S9. Rivera M*, et al*. (1998) The Reduction Potential of Cytochrome b5 Is Modulated by Its Exposed Heme Edge. *Biochemistry* 37(6):1485-1494.

S10. Schenkman JB & Jansson I (2003) The many roles of cytochrome b5. *Pharmacology & Therapeutics* 97(2):139-152.

S11. Zhang D, Wilson GS, & Niki K (1994) Electrochemistry of adsorbed cytochrome c3 on mercury, glassy carbon, and gold electrodes. *Analytical Chemistry* 66(22):3873-3881.
